# 

**Published:** 1894-12

**Authors:** 


### The Library of the Bristol Medico-Chirurgical Society.

---

At the Annual Meeting held on October 10th, 1894, Mr. L. M. Griffiths, the Honorary Librarian, read the following Report:—

Since the last Annual Meeting 481 volumes have been added to the Library, which now consists, including 424 duplicates, of 3,776 volumes.

The donors during the twelve months have been: Mr. J. W. Arrowsmith, The Dowager Lady Bowman, Dr. J. Broom, Dr. R. E. Burges, the Council of the Clinical Society, Mr. G. G. Corbould, Mr. W. Duncan, Dr. F. H. Edgeworth, Mr. J. Ewens, Mr. J. Fawn, Messrs. George's Sons, Mr. L.M. Griffiths, Mr. W.H. Harsant, Mr. F. P. Lansdown, Dr. C. Martin, Mr. Augustin Prichard, Dr. A. B. Prowse, the Royal Academy of Medicine in Ireland, Dr. J. E. Shaw, Mr. E. J. Sheppard, Mr. Greig Smith, Dr. Shingleton Smith, Mr. F. W. Stoddart, the Surgeon-General United States Army, the Council of University College, Bristol, the Editors of the Pathological Report of University College, London, Mr. Algernon Warren, Mr. W. Roger Williams, and Messrs. J. Wright & Co.

The combined arrangement with University College works well. The authorities of the College bestow much thoughtfulness on the ways of extending the usefulness of the Library. The services of the Clerk have been reinforced by those of an assistant, and one of these is always in the Library to attend to the wants of readers. Since my last Report the greater portion of the libraries of the Infirmary and Hospital, and of the Medical Section of the Museum and Library have been transferred to the Medical School, and members of the Society have now access to more than 13,000 volumes, and can take out on loan the duplicates, of which there are a large number. The number of current periodicals regularly received in the Library is over 120.

---

*The following donations have been received since the publication of the list in September:*

*November* 30*th*, 1894.

| | |
|---|---|
| T. F. Edgeworth (1) ... ... ... ... ... ... | 113 volumes. |
| L. M. Griffiths (2) ... ... ... ... ... ... ... | 1 volume. |
| A. J. Harrison, M.B. (3) ... ... ... ... ... ... ... | 83 volumes. |
| Augustin Prichard (4) ... ... ... ... ... ... | 2 ,, |
| R. Shingleton Smith, M.D. ... ... ... ... ... | 1 volume. |
| The Council of University College, Bristol (5) ... | 1 ,, |

Three framed pictures have been presented by Mr. T. F. Edgeworth, and two unframed by Mr. C. K. Rudge.

### FOURTEENTH LIST OF BOOKS.

The titles of books mentioned in previous lists are not repeated.

The figures in brackets refer to the figures after the names of the donors, and show by whom the volumes were presented. The books to which no such figures are attached have either been bought from the Library Fund or received through the *Journal*.

---

*Alcohol Question, The* ... ... ... ... ... ... ... ... ... ... (3) 2nd Ed. [N.D.]

**Anderson, J.** ... *Medical Nursing* (Ed. by Ethel F. Lamport) ... ... 1894

**Arbuthnot, J.** ... *An Essay concerning the Nature of Aliments* (1) 2nd Ed. 1732

**Atthill, L.** ... ... *Diseases Peculiar to Women* ... ... ... (3) 3rd Ed. 1875

**Baillie, M.** ... ... *Morbid Anatomy* ... ... ... ... ... ... (1) 8th Ed. 1833

**Bain, A.** ... ... *The Senses of the Intellect* ... ... ... (3) 3rd Ed. 1868

**Baker, J.** (Ed.) ... *New Guide to Bristol and Clifton* ... ... ... ... ... [1894]

**Balfour, G. W.** ... *The Senile Heart* ... ... ... ... ... ... ... ... ... 1894

**Black, D. C.** ... *The Urine in Health and Disease, and Urinary Analysis* 1895

**Bond, T. E.** ... *On Dental Medicine as connected with Study of Dental Surgery* ... ... ... ... ... ... ... ... (1) 2nd Ed. 1852

**Bowman, J. E.** ... *Medical Chemistry* ... ... ... ... ... ... (1) 2nd Ed. 1852

,, ... *Practical Chemistry* (3) 6th Ed. (Ed. by C. L. Bloxam) 1871

**Bowman, W.** ... *Lectures on the Eye* ... ... ... ... ... ... ... ... (3) 1849

**Brande, W. T.** ... *A Manual of Chemistry* ... ... ... ... (1) 4th Ed. 1836

**Brinton, W.** ... *Lectures on the Diseases of the Stomach* (3) 2nd Ed. 1864

,, ... *Intestinal Obstruction.* (Ed. by T. Buzzard) ... (3) 1867

**Brodhurst, B. E.** *On Club-Foot* ... ... ... ... ... ... ... ... ... ... (1) 1856

,, *Diseases of the Joints involving Anchylosis* (1) 3rd Ed. 1861

**Brodie, C. G.** ... *Dissections Illustrated* ... ... ... ... Part III. [1894]

**Bubis, G.** ... ... *Sperminum-Poehl in chemischer, physiologischer und therapeutischer Beziehung* ... ... ... ... ... ... ... 1894

**Bucknill and D. H. Tuke, J. C.** *A Manual of Psychological Medicine* (3) 2nd Ed. 1862

**Carpenter, R. L.** (Ed.) *Memoirs of the Life of the Rev. Lant Carpenter* (1) 1842

**Carpenter, W. B.** *A Manual of Physiology* ... ... ... ... (1) 2nd Ed. 1851

**Celsus, A. C.** ... *Medicine in Eight Books*—Latin and English. (Tr. by A. Lee) ... ... ... ... ... ... 2 vols. (3) 1831-36

**Chamberlaine, W.** *Tirocinium Medicum* ... ... ... ... ... ... ... ... (1) 1812

**Chambers, T. K.** ... *The Indigestions* ... ... ... ... ... ... ... ... (3) 1867

**Cheyne, W. W.** ... *The Treatment of Wounds, Ulcers, and Abscesses* ... ... 1894

**Churchill, J. F.** *Consumption and the Hypophosphites* ... ... ... (1) 1875

**Conquest, J. T.** ... *What is Homœopathy?* ... ... ... ... (1) 2nd Ed. 1859

**Cooper, S.** ... .. *The First Lines of the Practice of Surgery* (3) 5th Ed. 1826

,, *A Dictionary of Practical Surgery* ... ... (3) 7th Ed. 1838

**Corfe, G.** ... ... *A Popular Treatise on the Kidney* ... ... ... ... (1) 1839

*Cyclopædia, Everybody's Pocket* ... .. ... ... ... ... ... ... ... ... ... [N.D.]

**Da Costa, J. C.** ... *A Manual of Modern Surgery* ... ... ... ... ... ... 1894

**Dalton, J. C.** ... *Treatise on Human Physiology* ... ... (3) 2nd Ed. 1861

**Denman, T.** ... *Introduction to the Practice of Midwifery* (1) 7th Ed. (Ed. by C. Waller) ... ... ... ... ... ... ... 1832

**Dixon, J.** ... ... *Diseases of the Eye* ... ... ... ... ... (3) 2nd Ed. 1859

**Drewitt, F. D.** ... *On Gangrene of the Limbs following Typhoid Fever* ... 1894

**Earle, L.** ... ... *On Flooding after Delivery* ... ... ... ... ... (3) 1865

| | | |
|---|---|---|
| **Elliotson, J.** ... | *Lectures on the Theory and Practice of Medicine.* (Ed. by J. C. Cooke and T. G. Thompson) ... ... (1) | 1839 |
| **Erichsen, J. E.** ... | *On Concussion of the Spine* ... ... ... ... ... ... (3) | 1875 |
| **Falconer, W.** ... | *A Dissertation on the Influence of the Passions upon Disorders of the Body* ... ... ... ... ... ... ... (1) | 1788 |
| **Fenwick, W. S.**... | *The Dyspepsia of Phthisis* ... ... ... ... ... ... ... | 1894 |
| **Field, G. P.** ... | *Aural Surgery* ... ... ... ... ... ... ... ... ... ... (3) | 1876 |
| **Fothergill, J. M.** | *The Heart and its Diseases* ... ... ... ... ... ... (3) | 1872 |
| **Fox, J.** ... ... | *The Natural History of the Human Teeth* ... ... (1) | 1803 |
| ,, ... ... | *The History and Treatment of the Diseases of the Teeth, the Gums, etc.* ... ... ... ... ... ... ... ... ... (1) | 1806 |
| **Fresenius, C. R.**... | *Instruction in Chemical Analysis* (Quantitative). (Ed. by J. L. Bullock) .. ... ... ... ... ... ... ... (3) | 1846 |
| ,, | *Instruction in Chemical Analysis* (Qualitative). (Ed. by J. L. Bullock)... ... ... ... ... ... (3) 3rd Ed. | 1850 |
| **Gaine, C.** ... ... | *On Certain Irregularities of the Teeth* ... ... ... (1) | [N.D.] |
| **Gamgee, J. S.** ... | *Researches in Pathological Anatomy and Clinical Surgery* (1) | 1856 |
| ,, ... | *Amputation at the Hip-Joint* ... ... ... ... (3) | 1865 |
| **Garrod, A. B.** ... | *The Essentials of Materia Medica and Therapeutics* (3) 2nd Ed. | 1864 |
| ,, ... | *Gout and Rheumatic Gout* ... ... ... (3) 3rd Ed. | 1876 |
| **Gleason, E. B.** ... | *Essentials of the Diseases of the Ear* ... ... ... ... | 1894 |
| **Glenn, R. G.** ... | *A Manual of the Laws affecting Medical Men* ... (3) | 1871 |
| **Gray, S. F.**... ... | *Supplement to the Pharmacopœia.* (Ed. by T. Redwood) ... ... ... ... ... ... ... ... ... ... (1) | 1847 |
| **Gregory, J.**... ... | *Conspectus Medicinæ Theoreticæ* ... ... (1) 10th Ed. | 1836 |
| **Gregory, W.** ... | *Handbook of Organic Chemistry* ... ... (3) 4th Ed. | 1856 |
| **Hardwicke, H. J.** | *Alpine Climates for Consumption* ... ... ... ... ... | 1894 |
| **Hart, E.** ... ... | *The Nurseries of Cholera : Its Diffusion and its Extinction* | 1894 |
| **Hewitt, G.** ... ... | *Diseases of Women* ... ... ... ... ... ... ... ... (3) | 1863 |
| **Hodgson, J.** ... | *The Hunterian Oration* ... ... ... ... ... ... (2) | 1855 |
| **Hooper. R.**... ... | *Medical Dictionary* ... ... ... . . ... (1) 6th Ed. | 1831 |
| **Hovell, T. M.** ... | *Diseases of the Ear, etc.* ... ... ... ... ... ... ... ... | 1894 |
| **Howship, J.** ... | *Discrimination and Appearances of Surgical Disease* (1) | 1840 |
| **Hutton, A. W.** ... | *The Vaccination Question* ... ... ... ... ... ... ... ... | 1894 |
| **Inman, T.** ... ... | *The Restoration of Health* ... ... ... ... ... ... (3) | 1870 |
| **Jenner, W.**... ... | *Diphtheria*... ... ... ... ... ... ... ... ... ... ... (3) | 1861 |
| **Johnson. G.** ... | *Notes on Cholera* ... ... ... ... ... ... ... ... (3) | 1866 |
| **Jones, T. W.** ... | *Ophthalmic Medicine and Surgery* ... ... (1) 3rd Ed. | 1865 |
| **Jordan, F.** ... ... | *Surgical Inquiries* ... ... ... ... ... ... ... ... (3) | 1873 |
| **Kirkes, W. S.** ... | *Handbook of Physiology* (3) 6th Ed. (Ed. by W. M. Baker) | 1867 |
| **Laurie, J.** ... ... | *Epitome of the Homœopathic Domestic Medicine* ... (1) | 1853 |
| **Lawless, E. J.** ... | *First Aid to the Injured and Management of the Sick* ... | 1894 |
| **Lawrence, W.** ... | *A Treatise on Ruptures* ... ... ... ... (1) 5th Ed. | 1838 |
| **MacFarlane, A.**... | *A Clinical Manual* ... ... ... ... ... ... ... ... ... | 1894 |
| **Maddock, A. B.**... | *Pulmonary Consumption, Bronchitis, Asthma, etc.* (1) 4th Ed. | 1851 |
| **Manton, W. P.** ... | *Syllabus of Lectures on Human Embryology* ... ... ... | 1894 |
| **Manzaneque, M.**. | *Caracteristica terapêutica de las Aquas termales de La Garriga* ... ... ... ... ... ... ... ... ... ... ... ... | 1894 |
| **Merei, A. S.** ... | *On the Disorders of Infantile Development and Rickets* (1) | 1855 |
| **Miller, J.** ... ... | *The Principles of Surgery* ... ... ... (3) 3rd Ed. | 1853 |

| | | |
|---|---|---|
| **Moullin, C. W. M.** | *Enlargement of the Prostate* ... ... ... ... ... ... ... | 1894 |
| **Muskett, P. E.** ... | *Prescribing and Treatment in the Diseases of Infants and Children*... ... ... ... ... ... ... ... ... 3rd Ed. | 1894 |
| **Nodd, H. M.** ... | *Chemical Manipulation and Analysis, Qualitative and Quantitative* ... ... ... ... ... ... (3) [2nd Ed.] | 1852 |
| **Norris, R.** ... ... | *On the Aggregation of Blood Corpuscles* ... ... (3) | 1870 |
| ,, ... ... | *The Physiology and Pathology of the Blood* ... ... (3) | 1882 |
| **Osler, W.** ... ... | *On Chorea and Choreiform Affections* ... ... ... ... | 1894 |
| *Owen's Conspectus; or Student's Remembrancer*... | ... ... ... ... ... ... (1) | 1868 |
| **Page, H. W.** ... | *Injuries to the Spine and Spinal Cord* ... (3) 2nd Ed. | 1885 |
| **Parker, A.** ... ... | *Artificial Teeth* ... ... ... ... .. ... (3) 3rd Ed. | 1876 |
| **Parkes, S.** ... ... | *The Chemical Catechism* ... ... ... ... (1) 4th Ed. | 1810 |
| **Pemberton, O.** ... | *Clinical Illustrations of Various Forms of Cancer* (3, 4) | 1867 |
| **Pepper, A. J.** ... | *Elements of Surgical Pathology* ... ... ... 4th Ed. | 1894 |
| **Phillips, R.** ... | *Translation of the Pharmacopœia of R.C.P., Lond.* 1836 (1) 3rd Ed. | 1838 |
| **Pole, T.** ... ... | *The Anatomical Instructor* ... ... ... ... ... (3) | 1790 |
| **Potârcă, J.** ... ... | *Sur l'Oesophagotomie intrathoracique par le médiastin postérieur* ... ... ... ... ... ... ... ... | 1894 |
| **Purdy, C. W.** ... | *Practical Uranalysis and Urinary Diagnosis*... ... ... | 1894 |
| **Quain, J.** ... ... | *Elements of Anatomy* ... ... ... ... (1) 4th Ed. | 1837 |
| **Raymond, J. H.** | *A Manual of Human Physiology* ... ... ... ... ... | 1894 |
| **Reece, R.** ... ... | *The Medical Guide* ... ... ... ... ... ... ... (1) | 1804 |
| **Reed, D. M.** ... | *On Fever, Plague, Cholera Contagion, &c.* ... ... (3) | 1846 |
| **Reid, D. B.** ... | *Text-book for Students of Chemistry* ... (1) 2nd Ed. | 1836 |
| **Roberts, F. T.** ... | *The Theory and Practice of Medicine* ... ... 9th Ed. | 1894 |
| **Robinson, J.** ... | *The Surgical, Mechanical and Medical Treatment of the Teeth* ... ... ... ... ... ... ... ... ... (1) | 1846 |
| **Rohé, G. H.** ... | *Text-book of Hygiene* ... ... ... ... ... 3rd Ed. | 1894 |
| **Rumsey, H. W.**... | *Essays on State Medicine* ... ... ... ... ... ... (4) | 1856 |
| **Ryan, M.** ... ... | *Manual of Medical Jurisprudence and State Medicine* (1) 2nd Ed. | 1836 |
| **Scarpa, A.** ... ... | *A Treatise on the Anatomy, Pathology and Surgical Treatment of Aneurism.* (Tr. by J. H. Wishart) (1) | 1808 |
| **Smith, E.** ... ... | *Consumption: its Early and Remediable Stages* ... (1) | 1862 |
| ,, ... ... | *On Foods* ... ... ... ... ... ... ... (1) 3rd Ed. | 1874 |
| **Smith, J.** ... ... | *Hand-Book of Dental Anatomy and Surgery* ... (1, 3) | 1864 |
| **Solomon, J. V.** ... | *Tension of the Eyeball and Glaucoma, &c.* ... ... (3) | 1865 |
| **Stevens, J.**... ... | *Medical Reform*... ... ... ... ... ... (1) 3rd Ed. | 1848 |
| **Swan, R. L.** ... | *Diseases and Deformities of the Spine* ... ... ... ... | 1894 |
| **Tanner, T. H.** ... | *Diseases of Infancy and Childhood* ... ... ... ... (3) | 1858 |
| ,, ... | *The Practice of Medicine* ... ... ... ... (3) 5th Ed. | 1865 |
| **Taylor, A. S.** ... | *On Poisons* .. ... ... ... ... ... ... ... (1) | 1848 |
| **Thomas, W. H.** ... | *Surgical and Descriptive Anatomy of the Bones, Ligaments, and Joints* ... ... ... ... ... ... ... ... ... (1) | 1834 |
| **Thompson, Sir H.** | *Diseases of the Prostate* ... ... ... ... ... ... (1) | 1861 |
| ,, | *Clinical Lectures on Diseases of the Urinary Organs* (3) 2nd Ed. | 1869 |
| **Thomson, A. T.** ... | *The London Dispensatory*... ... ... ... (1) 4th Ed. | 1826 |
| **Thorowgood, J. C.** | *Asthma and Chronic Bronchitis* ... ... ... ... ... | 1894 |
| **Tod, D.** ... ... | *The Anatomy and Physiology of the Organ of Hearing* (3) | 1832 |

| | | |
|---|---|---|
| **Toynbee, J.... ...** | *The Diseases of the Ear* ... ... ... ... ... ... ... (1) | 1860 |
| **Tuke, J. C. Bucknill and D. H.** | *A Manual of Psychological Medicine* (3) 2nd Ed. | 1862 |
| **Tweedie, A. ...** | *A System of Practical Medicine.* Vol. I. ... ... (3) | 1840 |
| **Virchow, R. ...** | *Cellular Pathology.* (Tr. by F. Chance) ... ... (3) | 1860 |
| **Walker, S. ... ...** | *A Treatise on Nervous Diseases* ... ... ... ... (1) | 1796 |
| **Watson, P. H. ...** | *Excision of the Knee-Joint* .. ... ... ... ... (1) | 1867 |
| **Watt, J. J.... ...** | *An Encyclopedia of Surgery, Medicine, etc.* ... ... (1) | 1806 |
| **West, C. ... ...** | *Diseases of Infancy and Childhood* ... ... (3) 4th Ed. | 1859 |
| **Whitlaw, C. ...** | *A Treatise on Inflammation, Fever, Cancer, etc.* ... (1) | 1831 |
| **Williams, P. W....** | *Diseases of the Upper Respiratory Tract* ... ... ... | [1894] |
| **Williams, W. R....** | *Diseases of the Breast* ... ... ... ... ... ... ... ... | 1894 |
| **Wilson, G. ... ...** | *Inorganic Chemistry* (1) [3rd Ed.] (Ed. by S. Macadam) | [1866] |
| **Withington, E. T.** | *Medical History from the Earliest Times* ... ... ... | 1894 |
| **Wright, W. ...** | *On the Varieties of Deafness and Diseases of the Ear* (1) | 1829 |
| **Wyllie, J. ... ...** | *The Disorders of Speech* ... ... ... ... ... ... ... | [1894] |

### TRANSACTIONS, REPORTS, JOURNALS, &c.

| | | |
|---|---|---|
| American Journal of Dental Science, The ... ... | (1) Vols. VI., VII. | 1856-57 |
| American Journal of Obstetrics, The ... ... ... ... ... | Vol. XXIX. | 1894 |
| American Journal of Ophthalmology, The ... ... ... ... | Vol. X. | 1893 |
| Annual of the Universal Medical Sciences. 5 Vols. ... ... ... ... | | 1894 |
| Asclepiad, The ... ... ... ... ... ... ... ... ... ... | Vol. X. | 1893 |
| British Gynæcological Journal, The ... ... ... ... ... | Vol. IX. | 1893 |
| Buffalo Medical and Surgical Journal ... ... ... ... | Vol. XXXIII. | 1894 |
| Calendar of University College, Bristol ... ... ... ... ... ... | (5) | 1894 |
| Cincinnati Lancet-Clinic, The ... ... ... ... ... ... | Vol. LXXI. | 1894 |
| Clinical Society of London, Transactions of the ... ... | Vol. XXVII. | 1894 |
| Dental Review, The ... ... ... ... ... ... ... ... | (1) Vols. IV., V. | 1862-63 |
| Glasgow Medical Journal, The ... ... ... ... ... ... | Vol. XLI. | 1894 |
| Hospital, The ... ... ... ... ... ... ... ... ... ... ... | Vol. XVI. | 1894 |
| Hospitals— | | |
| Johns Hopkins Hospital Reports, The ... ... | Vol. IV., No. 6 | 1894 |
| Westminster Hospital Reports, The... ... ... ... | Vol. VIII. | 1893 |
| Journal of the American Medical Association, The ... ... | Vol. XXII. | 1894 |
| Lancet, The ... ... (1) Vol. II. for 1827-28; (1) Vol. I. for 1828-29; (1) Vol. I. for 1830-31 | | |
| London and Provincial Medical Directory, The ... | (1) 1848, 1858, | 1861 |
| London Medical Gazette, The ... ... ... ... | (1) Vols. III.—VI. | 1829-30 |
| Medical and Surgical Reporter, The ... ... ... ... ... | Vol. LXX. | 1894 |
| Medical Directory, The ... ... ... ... ... .. ... ... ... | (1) | 1873 |
| Medical List, The ... ... ... ... ... ... ... ... ... ... | (1) | 1857 |
| Medical Register, The ... ... ... ... ... ... ... ... ... | (1) | 1887 |
| Medico-Chirurgical Review, The ... ... ... ... ... ... ... | (1) | 1822-23 |
| Montreal Medical Journal, The ... ... ... ... ... ... | Vol. XXII. | 1894 |
| Odontological Society of London, Transactions of the, 1856-57 ... | (1) | 1858 |
| Ophthalmological Society of the United Kingdom, Transactions of the | Vol. XIV. | 1894 |
| Pharmaceutical Journal and Transactions ... ... | (1) Vols. IV.—VI. | 1845-47 |
| Polytechnic Review and Magazine, The ... ... | [Vol. II. of 1844] (1) | 1844 |
| Quarterly Medical Journal, The ... ... ... ... ... ... | Vol. II. | 1894 |

### LIST OF SUBSCRIBERS

TO

### The Bristol Medico-Chirurgical Journal.

*DECEMBER*, 1894.

---

### England.

### BERKSHIRE.

READING.
F. A. Hill

### BUCKINGHAMSHIRE.

WINSLOW.

W. H. Walter

### CHESHIRE.

CHESTER.
R. E. Burges

EGREMONT.
T. W. A. Napier

NORTHWICH.
P. T. Tolputt

MACCLESFIELD.
W. R. Etches

### CORNWALL.

NEW QUAY.
A. Hardwick

PADSTOW.
H. F. Marley

PENZANCE.
W. S. Bennett
J. A. Fox
J. Symons

PORT ISAAC.
R. J. George

ROCHE.

W. R. Goodfellow

ST. AUSTELL.
W. Mason

TORPOINT.
S. G. Vinter

TRURO.
W. A. G. Laing.

### DEVONSHIRE.

BABBACOMBE.
T. Finch

BARNSTAPLE.
C. H. Gamble
J. Harper
M. Jackson
F. Penny

BRIXHAM.
G. C. Searle

BROAD CLYST.
J. W. Sandoe
J. Somer

BUDLEIGH SALTERTON.
H. F. Semple

CHAGFORD.
A. D. Hunt

CHERITON FITZPAINE.
G. L. Thorne

CHUDLEIGH.
C. L. Cunningham

DARTMOUTH.
R. B. Searle

DAWLISH.
A. de W. Baker

DEVONPORT.
F. E. Row

EXETER.
J. M. Ackland
A. G. Blomfield
H. Davy
P. M. Deas
E. J. Domville
W. Gordon
A. W. Kempe
R. V. Solly
E. B. Steele-Perkins
L. H. Tosswill

HONITON.
T. W. Shortridge

MILLBROOK.
A. B. Cheves

MORETON HAMPSTEAD.
E. H. Openshaw

NEWTON ABBOT.
J. Culross
E. Haydon

### DEVONSHIRE (*Continued*).

PAIGNTON.

J. Alexander

PLYMOUTH.

G. F. Aldous
R. H. Clay
E. L. Fox
R. H. Lucy
Medical Society
E. B. Thomson
W. L. Woollcombe

PLYMPTON.

C. Aldridge
W. D. Stamp

ST. MARY CHURCH.

T. Finch

TORQUAY.

H. Humphreys
W. Odell
R. Pollard
W. Powell
J. T. Thomas
C. H. Wade

UFFCULME.

F. Morgan

WILLAND.

H. E. Tracey

WINKLEIGH.

J. H. Norman

### DORSETSHIRE.

BEAMINSTER.

T. P. Daniel
F. P. Kitson

DORCHESTER.

F. B. Fisher
P. W. MacDonald

GILLINGHAM.

E. O. Cox

POOLE.

J. McNicoll

PORTLAND.

G. H. Lilley

STURMINSTER NEWTON

F. H. Hudson
J. C. Leach

WEYMOUTH.

J. M. Lawrie

WIMBORNE.

A. J. H. Crespi

### ESSEX.

NEWPORT.

W. A. Smith

### GLOUCESTERSHIRE.

BLAKENEY.

A. M. Irwin

BRISTOL.

W. R. Ackland
G. Adams
J. B. Adams
G. F. Atchley
W. M. Barclay
T. G. L. Baretti
B. J. Baron
A. E. Blacker
E Blacker
A. F. Blagg
E. C. Board
J. G. Boyce
C. W. J. Brasher
J. Broom
E. F. H. Burroughs
J. P. Bush
J. Cameron
A. Carr
T. M. Carter
W. F. Carter
W. L. Christie
J. M. Clarke
E. W. Coathupe
R. W. Coe
T. V. Coker
T. J. Colman
H. Cook
G. G. Corbould
W. Cotton
F. R. Cross
J. Dacre
D. S. Davies
H. F. Devis
N. C. Dobson
W. Dowson
E. L. W. Dunbar
F. H. Edgeworth
W. Elder
C. Elliott
J. Ewens
R. G. Fendick
J. L. Firth
T. Fisher
E. L. Fox
W. J. Fyffe
A. N. G. Gibbs
J. Gill
G. A. Gloag
C. Gordon
W. G. Grace
J. S. Griffiths
L. M. Griffiths
C. D. G. Hailes
A. H. Haines
A. J. Harrison
W. H. Harsant
A. G. Hayman
C. A. Hayman
J. C. Heaven
C. K. C. Herapath
H. E. Hetling
H. Hill
T. Hill
W. J. Hill
F. F. Hills
General Hospital
G. A. Imlay
A. Jamison
D. G. Johnston
T. B. Kelly
F. P. Lansdown
R. G. P. Lansdown
A. E. A. Lawrence
H. Lawrence
E. L. Lees
W. Ligertwood
Museum and Library
W. E. Lloyd
P. W. McCrea
H. W. G. Macleod
W. T. Maddison
H. Marshall
C. E. Matthews
T. O. Mayor
J. S. Metford
J. Milne
H. F. Mole
C. A. Morton
G. T. Myles
B. G. Neale
W. N. Nevill
C. Newman
W. H. C. Newnham
T. D. Nicholson
J. A. Norton
C. O'Brien
A. Ogilvy
H. L. Ormerod
G. S. Page
G. Parker
J. H. Parry
G. C. P. Pauli
A. W. Peake
W. J. Penny
C. F. Pickering
A. E. H. Pinch
W. E. Pountney
J. J. Powell
A. Prichard
A. W. Prichard
J. E. Prichard
A. Pring
A. B. Prowse
B. M. H. Rogers

### GLOUCESTERSHIRE (*Continued*).

BRISTOL (*continued*).

W. B. Roué
C. K. Rudge
H. T. Rudge
J. E. Shaw
E. J. Sheppard
E. M. Skerritt
G. M. Smith
J. G. Smith
R. Smith
R. S. Smith
C. Steele
W. H. Stevens
W. M. Stevens
F. W. Stoddart
J. Swain
J. G. Swayne
S. H. Swayne
W. C. Swayne
J. Taylor
W. C. Thompson
W. J. Tivy
E. N. Tribe
H. Waldo
C. H. Walker
C. H. Wallace
J. H. Wathen
T. Webster
J. Wilding
P. W. Williams
H. W. Windsor-Aubrey
C. Wintle

CHELTENHAM.

A. Duke
Medical Library
C. E. F. Mouat-Biggs
G. A. Peake
L. Winterbotham

CHIPPING SODBURY.

A. Grace

CINDERFORD.

W. C. Heane

CIRENCESTER.

W. R. Cossham
O. H. Fowler
C. Mackinnon

COLEFORD.

L. B. Trotter

DOWNEND.

H. Skelton

FISHPONDS.

W. Brown

GLOUCESTER.

R. W. Batten
T. Edis
J. G. Soutar

HAMBROOK.

E. Crossman
W. F. B. Eadon

KINGSWOOD.

H. Grace

LYDNEY.

W. P. Kennedy

MORETON-IN-MARSH.

L. K. Yelf

PARKEND.

W. C. Halpin

REDFIELD.

J. W. Rodgers

STAPLE HILL.

W. Murray

STAPLETON.

H. T. S. Aveline
H. A. Benham

STOKE BISHOP.

W. J. K. Millard
C. E. Wedmore

STONEHOUSE.

G. Carolin
G. T. B. Watters

STOW-ON-THE-WOLD.

E. Dening

STROUD.

A. S. Cooke
E. A. Howsin
F. W. Storry

TETBURY.

W. Wickham

THORNBURY.

E. M. Grace
T. H. Taylor
L. H. Williams

WESTBURY-ON-TRYM.

W. Duncan
A. Harvey
H. Ormerod

WINTERBOURNE.

R. Eager

WOTTON-UNDER-EDGE.

D. H. Forty

### HAMPSHIRE.

ALDERSHOT.

J. C. Barr

ALTON.

W. L. P. Bevan

BOURNEMOUTH.

J. S. Dickie
D. C. Embleton
J. G. Harsant
J. Horsfall
W. E. Shorland
A. J. Turner

CARISBROOKE.

J. Groves.

DROXFORD.

A. H. Goodwyn

FLEET.

H. Wilcox

LYMINGTON.

J. Rendall

ST. MARY BOURNE.

A. W. Riddell

SOUTHAMPTON.

W. R. Y. Ives

SOUTHSEA.

J. W. Cousins

VENTNOR.

E. R. Woodford

### HEREFORDSHIRE.

HEREFORD.

W. C. Whitfeld

### HERTFORDSHIRE.

ST. ALBAN'S.

A. H. Boys

### HUNTINGDONSHIRE.

ST. NEOT'S.

J. H. Hemming.

### KENT.

MAIDSTONE.

R. H. Norgate

### LANCASHIRE.

LIVERPOOL.

G. A. Hawkins-Ambler
Medical Institution
C. Williams

POULTON-LE-FYLDE.

P. H. Day

PRESTON.

J. E. Garner

### MIDDLESEX.

LONDON.

A. E. Barker
J. Berry
J. G. Davey
J. F. Evans
T. C. Fox
J. M. Granville
G. W. Grote
H. W. M. Hayes
M. Handfield-Jones
W. H. A. Jacobson
H. M. Jones
T. L. Laxton
J. Lister
E. J. Maclean
J. Macready
A. L. Marshall
W. Pippette
F. T. Roberts
M. A. Ruffer
W. J. Seward
D. Sinclair
N. Smith
J. A. Watson
F. J. Wethered
G. S. Woodhead

TOTTENHAM.

W. H. Plaister

### MONMOUTHSHIRE.

BLAENAVON.

A. B. Avarne.

NEWPORT.

W. Basset
C. B. Gratte
H. R. Hudson
O. E. B. Marsh
A. G. Thomas
H. E. Williams

PONTYPOOL.

S. B. Mason

RHYMNEY.

T. H. Redwood

RISCA.

G. B. Robathan

TREDEGAR.

G. A. Brown

### NOTTINGHAMSHIRE.

NOTTINGHAM.

C. B. Taylor

### OXFORDSHIRE.

HENLEY-ON-THAMES.

J. Lidderdale

OXFORD.

R. W. Doyne
H. P. Symonds

### SOMERSETSHIRE.

BATH.

G. A. Bannatyne
A. B. Brabazon
C. S. W. Cobbold
T. Cole
F. S. Cowan
S. Craddock
W. M. Ellis
A. E. W. Fox
H. W. Freeman
H. F. A. Goodridge
T. B. Goss
F. P. Hoblyn
H. C. Hopkins
L. J. King
F. Lace
H. Lane
T. P. Lowe
R. J. H. Scott
L. H. Walsh
L. A. Weatherly

BECKINGTON.

W. G. Evans

BRIDGWATER.

F. J. C. Parsons
R. H. F. Routh
J. B. Sincock

BRISLINGTON.

B. B. Fox
C. H. Fox

BURNHAM.

F. C. Berry
A. W. C. Peskett

CHEDDAR.

R. W. Statham

CHEW MAGNA.

E. T. Hale

CHILTON POLDEN.

G. Campbell

CLEVEDON.

T. Davis
J. A. F. Sawyer
S. Skinner

CREWKERNE.

E. J. Cave
W. W. Webber

CURRY RIVEL.

R. H. Vereker

### SOMERSETSHIRE *(Continued).*

FRESHFORD.
C. E. S. Flemming

FROME.
F. Parsons
J. M. Rattray

GLASTONBURY.
J. A. Bright

HIGHBRIDGE.
W. R. Hadwen

ILMINSTER.
C. Munden

KEYNSHAM.
C. Harrison
G. G. D. Willett

LANGPORT.
J. Morgan

MIDSOMER NORTON.
A. Waugh
A. H. Whicher

MILVERTON.
C. Randolph

NORTH PETHERTON.
C. F. Hawkins

PILL.
T. W. S. Morgan
R. A. Ross

PORTISHEAD.
W. Monckton
C. A. Wigan

SHEPTON MALLET.
J. T. Hyatt

SOUTH PETHERTON.

S. W. MacIlwaine

TAUNTON.
H. J. Alford
Taunton Hospital
J. A. Macdonald
H. T. Rutherford

TEMPLE CLOUD.
T. Martin

TIMSBURY.
F. Woods

WERTON-ON-AVON.
J. Wigmore

WEDMORE.
J. Ford
R. P. Tyley

WELLINGTON.
J. Meredith

WELLS.

F. J. B. Bateman

W. Fairbanks

A. L. Wade

WESTON-SUPER-MARE.
G. E. Alford
C. P. Crouch
G. B. Fraser
T. C. Grey
E. F. Martin
W. H. G. Phelps
G. F. Rossiter
R. Roxburgh

WORLE.
F. St. J. Kemm

WRINGTON.
A. H. Benson
H. W. Collins

YEOVIL.
P. S. H. Colmer

### STAFFORDSHIRE.

STAFFORD.
J. Scott

### SURREY.

EPSOM.
R. Davis

ESHER.
J. B. Fry

VIRGINIA WATER.
S. R. Philipps

REDHILL.
C. M. Jessop

### SUSSEX.

ST. LEONARD'S-ON-SEA.
W. H. Spencer

### WARWICKSHIRE.

BIRMINGHAM.
L. Tait

LEAMINGTON.
A. W. W. Hoffman.

### WILTSHIRE.

BRADFORD-ON-AVON.
J. Beddoe

BURBAGE.
A. E. N. Browne

CALNE.
W. S. Batten
W. A. Hayes

DEVIZES.
T. B. Anstie
C. Eddowes
A. M. Gray
H. J. Mackay

FOVANT.
C. Clay

MALMESBURY.
R. Kinneir

MERE.
J. McElfatrick

PEWSEY.
H. Colman

SALISBURY.
H. Coates
L. S. Luckham

SWINDON.
E. D. Duffett
W. Howse
J. C. Maclean

WARMINSTER.
R. L. Willcox

### YORKSHIRE.

HARROGATE.
A. O. Jones

HULL.
J. B. Close

SHEFFIELD.
S. Snell

### Ireland.

### ANTRIM.

BELFAST.
J. W. Browne
J. A. Lindsay

### Scotland.

### EDINBURGHSHIRE.

EDINBURGH.
J. W. Ballantyne
Y. J. Pentland

### Wales.

### BRECONSHIRE.

BRECON.
A. Whyte

BRYNMAWR.
G. H. Browne

SENNY BRIDGE.
W. R. Jones

TALGARTH.
W. Howells

### CARMARTHENSHIRE.

CARMARTHEN.
R. G. Price

FERRYSIDE.
P. Williams

LLANELLY.
S. J. Roderick
D. J. Williams

### CARNARVONSHIRE.

BETHESDA.
J. William

### GLAMORGANSHIRE.

ABERAMAN.
J. B. James

ABERDARE.
E. Jones

CAERPHILLY.
J. Lewellyn

CARDIFF.
E. T. Collins
W. T. Edwards
P. R. Griffiths
J. Milward
D. R. Paterson
J. A. Phillips
W. Price
A. Rees
A. Sheen
Medical Society
J. L. Thomas
H. R. Vachell
J. Williams

COWBRIDGE.
T. R. Hamlen-Williams

DERI.
D. Kent-Jones

MAESTEG.
W. H. Thomas

MERTHYR TYDVIL.
W. W. Jones
C. E. G. Simons
J. L. W. Ward

PENARTH.
R. F. Nell

PORTH.
H. N. Davies

SWANSEA.

E. Davies

R. C. Elsworth

C. A. Griffiths

TREHARRIS.
W. W. Leigh

### PEMBROKESHIRE.

FISHGUARD.
H. L. Swete

HAVERFORDWEST.
E. P. Phillips

PENALLY.
B. C. Kendall

### Channel Islands.

GUERNSEY.
N. P. Webster

### Belgium.

COURTRAI.
Dr. Lauwers

### Italy.

GENOA.

S. Bramonti

NAPLES.

C. Scimonelli

### Switzerland.

DAVOS-PLATZ.
W. R. Huggard

### Victoria.

BRADFORD.
G. H. Skinner

### Cape Colony.

FORT BEAUFORT.
J. Shaw

### Egypt.

ALEXANDRIA.
J. Mackie

### United States.

CINCINNATI.
Cincinnati Hospital

COLORADO.
S. E. Solly

NEW YORK.
J. B. Jackson

### INDEX.

Abdominal section, The after-treatment of cases of—Dr. C. Martin (*Review*), 324.
Abdominal surgery, Text-book of—S. and G. E. Keith (*Review*), 315.
Aitchison, Dr. R. S.—Medical handbook (*Review*), 46.
Alcohol and public health—Dr. J. J. Ridge (*Review*), 138.
Ambulance card—Dr. L. Morgan (*Review*), 59.
Amenorrhœa, Pigmentation in—Dr. A. E. A. Lawrence, 107.
American Surgical Association, Transactions of the (*Review*), 259.
Amygdale, Sur les abcès chroniques enkystés de l'—Dr. E. Peyrissac (*Review*), 134.
Annual of the universal medical sciences (*Review*), 319.
Antisepsis in the treatment of eczema, 247.
Antiseptics, Disinfectants and—Dr. E. T. Wilson (*Review*), 59.
Antiseptics in midwifery, The use of—Dr. R. Boxall (*Review*), 258.
Antitoxic serum, Use of, 241.
Antitoxin in the treatment of diphtheria, 306.
Appendicitis—Dr. J. Swain, 9.
Appendix, Pathology of the vermiform—Dr. T. N. Kelynack (*Review*), 49.
Archives of pediatrics, 143.
Association, British Medical—Bristol meeting, 161.
Association, British Medical—Bath and Bristol branch, 68.
Australia, The art of living in—P. E. Muskett (*Review*), 255.

Ballantyne, Dr. J. W.—Teratologia (*Review*), 137.
Barclay, W. M.—Report on surgery, 37.
Baron, Dr. B. J.—Report on laryngology, 306.
"Bazin's legs," 246.
Bibliographie méthodique des maladies de l'enfance, 143.
Blackwell, Dr. E.—The human element in sex (*Review*), 257.
Bladder, Tumours of the, 114.
Boxall, Dr. R.—The use of antiseptics in midwifery (*Review*), 258.
Bramwell, Dr. B.—Atlas of clinical medicine (*Review*), 322.
Breast, So-called "duct cancer" of the—C. A. Morton, 25.
Brewis, Dr. N. T.—Outlines of gynæcological diagnosis (*Review*), 258.
Bright's disease, Influence of diet in, 30.
Bristol health report (*Review*), 262.
Brodie, C. G.—Dissections illustrated (*Review*), 131.
Bury, Dr. J. S.—Clinical medicine (*Review*), 309.

Calculi of the bladder, Introduction to the catalogue of the collection of—Sir H. Thompson (*Review*), 138.
Campbell, Dr. H.—Headache and other morbid cephalic sensations (*Review*), 256.
Cancer, sarcoma, &c.—Dr. J. J. Clarke (*Review*), 130.
Cancer by electricity, The healing of rodent—Dr. J. I. Parsons, (*Review*), 255.
Cancers and the cancer process—Dr. H. Snow (*Review*), 128.
Carbohydrates, The physiology of the—Dr. F. W. Pavy (*Review*), 314.
Carcinoma of the rectum, 243.
Cardiac asthenia, 113.
Carpenter, Dr. G.—Congenital affections of the heart (*Review*), 312.
Childhood, Diseases of—Dr. H. B. Donkin (*Review*), 124.
Children, Adherent pericardium in—Dr. T. Fisher, 93.
Children, An American text-book of the diseases of—Drs. L. Starr and T. S. Westcott [Eds.] (*Review*), 310.
Children, Diseases of—Dr. J. McCaw (*Review*), 321.
Chorea—Dr. O. Sturges (*Review*), 258.

Churchill, Dr. J. F.—The stœchiological cure of consumption (*Review*), 133.

Clarke, Dr. J. J.—Cancer, sarcoma, &c. (*Review*), 130.

Clarke, Dr. J. M.—Report on medicine, 30; Somnambulism, 81.

Clinical museum and journal of proceedings, A descriptive catalogue of the—J. Hutchinson (*Review*), 316.

Clinical Society of London, Transactions of the (*Review*), 139.

Comparative pathology—Dr. A. J. Harrison, 273.

Congenital affections of the heart—Dr. G. Carpenter (*Review*), 312

Consumption, The hygienic prevention of—Dr. J. E. Squire (*Review*), 140.

Consumption, The stœchiological cure of—Dr. J. F. Churchill (*Review*), 133.

Corfield, Dr. W. H. — Dwelling houses (*Review*), 321.

Cotterell, E.—Syphilis (*Review*), 130.

Curvature of the spine, Rotary-lateral, 37.

Cysts of unusual origin—C. A. Morton, 232.

Death from traumatic fever, The physiology of—Dr. J. D. Malcolm (*Review*), 254.

Dermatology, Report on—Dr. H. Waldo, 245.

Diabetes, 238.

Diagnosis, Outlines of gynæcological—Dr. N. T. Brewis (*Review*), 258

Diarrhœa, Treatment of, 35.

Diet charts (*Review*), 59.

Digestion, Gastric, 33.

Diphtheria and its treatment—Dr. B. R. Martin (*Review*), 141.

Diphtheria, Antitoxin in the treatment of, 306.

Disinfectants and antiseptics—Dr. E. T. Wilson (*Review*), 59.

Dissections illustrated—C. G. Brodie (*Review*), 131.

Donald, Dr. A.—An introduction to midwifery (*Review*), 256.

Donkin, Dr. H. B.—Diseases of childhood (*Review*), 124.

Doyen, E.—Traitement chirurgical des affections inflammatoires et néoplasiques de l'utérus et de ses annexes (*Review*), 48.

"Duct cancer" of the breast, So-called—C. A. Morton, 25.

Dukes, Dr. C.—On the features which distinguish epidemic roseola from measles and from scarlet fever (*Review*), 261.

Dwelling houses—Dr. W. H. Corfield (*Review*), 321.

Eccles, Dr. A. S.—Sciatica (*Review*), 133.

Eczema, Antisepsis in the treatment of, 247.

Edgeworth, Dr. F. H.—A case of syringomyelia, 100.

Edinburgh hospital reports (*Review*), 51.

Educación, Hygiene de la (*Review*), 134.

Egyptian medicine, Ancient—Dr. J. Finlayson (*Review*), 138.

Electricity, The healing of rodent cancer by—Dr. J. I. Parsons (*Review*), 255.

Electrothérapie gynécologique, Travaux d' (*Review*), 319.

Enteric fever, 112.

Enteritis, Desquamative, 113.

Epilepsy by subcutaneous injections of testicular fluid, Treatment of, 36.

Ergot of rye as an oxytocic—Dr. J. G. Swayne, 225.

Faulkner, A.—A guide to the public medical services (*Review*), 142.

Ferguson, Drs. F. W. N. Haultain and J. H.—Handbook of obstetric nursing (*Review*), 324.

Filariosis, De la—Dr. M. Font (*Review*), 321.

Finlayson, Dr. J.—Ancient Egyptian medicine (*Review*), 138.

Firebaugh, E. M.—The physician's wife and the things that pertain to her life (*Review*), 312.

Fisher, Dr. T.—Adherent pericardium in children, 93.

Font, Dr. M.—De la filariosis (*Review*), 321.

Forensic medicine and toxicology—Dr. J. D. Mann (*Review*), 54.

Fuchs, Dr. E.—Text-book of ophthalmology (*Review*), 122.

Gall-stones, 31.

Galton, Sir D.—Healthy hospitals (*Review*), 127.

Garrigues, Dr. H. J.—A text-book of the diseases of women (*Review*), 250.

Gastric digestion, 33.

Genito-urinary diseases, Lectures on—Dr. J. C. O. Will (*Review*), 257.

Goodhart, Dr. J. F.—On common neuroses (*Review*), 259.
Gorham, J.—Tooth extraction (*Review*), 58.
Griffiths, L. M.—Report on obstetrics, 119.
Guy's hospital reports (*Reviews*), 131, 323.
Gynæcological diagnosis, Outlines of—Dr. N. T. Brewis (*Review*), 258.
Gynécologique, Travaux d'électrothérapie, (*Review*), 319.
Gynecology, The student's handbook of (*Review*), 137.

Harrison, Dr. A. J.—Comparative pathology, 273.
Harrison, R.—Lectures on the surgical disorders of the urinary organs (*Review*), 126.
Harrogate, The mineral waters of—Dr. J. Liddell (*Review*), 138.
Harsant, W. H.—Report on otology, 43; Report on surgery, 242.
Hart, E.—On the use of opium in India (*Review*), 320.
Haultain and J. H. Ferguson, Drs. F. W. N.—Handbook of obstetric nursing (*Review*), 324.
Haultain, Dr. F. W. N.—A practical handbook of midwifery (*Review*), 142.
Hayes, W. A.—Case of myxœdema, 230.
Headache and other morbid cephalic sensations—Dr. H. Campbell (*Review*), 256.
Heart, Congenital affections of the —Dr. G. Carpenter (*Review*), 312.
Heart, Diseases of—Dr. W. B. Thorne (*Review*), 321.
Heart, Functional diseases of the, 298.
Hemorrhoids, 242.
Herman, Dr. G. E.—Difficult labour (*Review*), 251.
Hernia—Dr. T. H. Manley (*Review*), 253.
Higiene de la educación (*Review*), 134.
Hill, Dr. A.—The physiologist's note-book (*Review*), 50.
Horne, Dr. J. F.—Trephining in its ancient and modern aspect (*Review*), 311.
Hospital annual, Burdett's (*Review*), 135.
Hospitals, Healthy—Sir D. Galton (*Review*), 127.
Hudson, T. J.—The law of psychic phenomena (*Review*), 53.
Hutchinson, J.—A descriptive catalogue of the clinical museum and journal of proceedings (*Review*), 316.

India, On the use of opium in—E. Hart (*Review*), 320.
Infectious diseases—Dr. L. C. Parkes (*Review*), 259.
Intercolonial quarterly journal of medicine and surgery (*Review*), 263.
International congress of hygiene and demography, 154.
Intussusception—A. W. Prichard, 1.
Ireland, Transactions of the Royal Academy of Medicine in (*Review*), 259.
Iron in the animal body, Absorption of, 113.

Jenks memorial prize, 154.
Johns Hopkins hospital reports (*Reviews*), 59, 136, 262.

Keith, S. and G. E.—Text-book of abdominal surgery (*Review*), 315.
Kelynack, Dr. T. N.—Pathology of the vermiform appendix (*Review*), 49.
Krafft-Ebing, D. R. von—Psychopathia sexualis (*Review*), 47.

Labour, Difficult—Dr. G. E. Herman (*Review*), 251.
Laryngology, Report on—Dr. B. J. Baron, 306.
Lateral sinus, Septic thrombosis of, 43.
Lawrence, Dr. A. E. A.—Pigmentation in amenorrhœa, 107.
Legg, J. W.—A guide to the examination of the urine (*Review*), 133.
Lewers, Dr. A. H. N.—A practical text-book of the diseases of women (*Review*), 58.
Library of the Bristol Medico-Chirurgical Society, 75, 152, 268, 330.
Library of the Surgeon-General's Office, United States Army, Index-catalogue of (*Review*), 136.
Liddell, Dr. J.—The mineral waters of Harrogate (*Review*), 138.
Lungs and pleuræ, Diseases of the—Dr. R. D. Powell (*Review*), 54.

McCaw, Dr. J.—Aids to the diagnosis and treatment of diseases of children (*Review*), 321.
MacIlwaine, S.W.—Cases of senile nerve degeneration resembling general paralysis, 294.
Malcolm, Dr. J. D.—The physiology of death from traumatic fever (*Review*), 254
Manley, Dr. T. H.—Hernia (*Review*), 253.

Mann, Dr. J. D.—Forensic medicine and toxicology (*Review*), 54.
Martin, Dr. B. R.—Diphtheria and its treatment (*Review*), 141.
Martin, Dr. C.—The after-treatment of cases of abdominal section (*Review*), 324.
Martin, Dr. E.—Essentials of minor surgery, bandaging, and venereal diseases (*Review*), 139.
Materia medica and therapeutics —Dr. J. V. Shoemaker (*Review*), 58.
Mathews' medical quarterly (*Review*), 56.
Medical annual (*Review*), 135.
Medical handbook—Dr. R. S. Aitchison (*Review*), 46.
Medical treatment, A manual of—Dr. I. B. Yeo (*Review*), 125.
Medicated baths in the treatment of skin diseases—Dr. L. Phillips (*Review*), 258.
Medicine, A text-book of the theory and practice of—Dr. W. Pepper [Ed.] (*Review*), 45, 129.
Medicine, Atlas of clinical—Dr. B. Bramwell (*Review*), 322.
Medicine, Clinical—Dr. J. S. Bury (*Review*), 309.
Medicine, Reports on—Dr. J. M. Clarke, 30; Dr. G. Parker, 235; Dr. R. S. Smith, 108, 297.
Middlesex hospital reports (*Review*), 325.
Midwifery, An introduction to—Dr. A. Donald (*Review*), 256.
Midwifery, A practical handbook of —Dr. F. W. N. Haultain (*Review*), 142.
Midwifery, The use of antiseptics in —Dr. R. Boxall (*Review*), 258.
Minor surgery, bandaging, and venereal diseases, Essentials of— —Dr. E. Martin (*Review*), 139.
Morgan, Dr. L.—Ambulance card (*Review*), 59.
Morris, M.—Diseases of the skin (*Review*), 141.
Morton, C. A.—Report on surgery, 114; So-called "duct cancer" of the breast, with the account of a case of large recurrent duct papilloma, 25; Two cysts of unusual origin, 232.
Moullin, Dr. C. W. M.—Sprains (*Review*), 324.
Moure, Dr. E. J.—De l'extraction des osselets dans l'otorrhée (*Review*), 320.
Mueller, Dr. A.—On snake-poison (*Review*), 51.
Murrell, Dr. W.—What to do in cases of poisoning (*Review*), 56.
Muskett, P. E.—The art of living in Australia (*Review*), 255.
Myxœdema—Dr. A. M. Wilson (*Review*), 140.
Myxœdema, Case of—W. A. Hayes, 230.

Nasal growths, Post—C. A. Parker (*Review*), 323.
Neuritis, Sciatic—R. Simpson (*Review*), 133.
Neuroses, On common—Dr. J. F. Goodhart (*Review*), 259.
New York Academy of Medicine, Transactions of the (*Reviews*), 57. 260.

Obstetric nursing, Handbook of—Drs. F. W. N. Haultain and J. H. Ferguson (*Review*), 324.
Obstetrics, Report on — L. M. Griffiths, 119.
Ophthalmology, Text-book of—Dr. E. Fuchs (*Review*), 122.
Opium in India, On the use of—E. Hart (*Review*), 320.
Otology, Aids to—W. R. H. Stewart (*Review*), 140.
Otology, Report on—W. H. Harsant, 43.
Otorrhée, De l'extraction des osselets dans l'—Dr. E. J. Moure (*Review*), 320.

Parker, C. A.—Post-nasal growths (*Review*), 323.
Parker, Dr. G.—Report on medicine, 235.
Parkes, Dr. L. C.—Infectious diseases (*Review*), 259.
Parsons, Dr. J. I.—The healing of rodent cancer by electricity (*Review*), 255.
Pathology, Comparative—Dr. A. J. Harrison, 273.
Pavy, Dr. F. W.—The physiology of the carbohydrates: their application as food and relation to diabetes (*Review*), 314.
Payne, Dr. J. F.—On seborrhœa (*Review*), 320.
Pepper, Dr. W. [Ed.]—A text-book of the theory and practice of medicine (*Review*), 45, 129.
Pericardium in children, Adherent —Dr. T. Fisher, 93.
Peyrissac, Dr. E.—Sur les abcès chroniques enkystés de l'amygdale (*Review*), 134.

Phillips, Dr. J.—Outlines of the diseases of women (*Review*), 52.
Phillips, Dr. L.—Medicated baths in the treatment of skin diseases (*Review*), 258.
Phthisis, Contagion in, 108.
Physician's wife and the things that pertain to her life, The—E. M. Firebaugh (*Review*) 312.
Physiologist's note-book, The—Dr. A. Hill (*Review*), 50.
Pigmentation in amenorrhœa—Dr. A. E. A. Lawrence, 107.
Pneumonia, 297.
Poisoning, What to do in cases of —Dr. W. Murrell (*Review*), 56.
Powell, Dr. R. D.—Diseases of the lungs and pleuræ, including consumption (*Review*), 54.
Pregnancy, Toxæmia of, 119; Vomiting in, 120.
Prichard, A. W.—Intussusception, 1.
Psychic phenomena, The law of—T. J. Hudson (*Review*), 53.
Psychopathia sexualis—D. R. von Krafft-Ebing (*Review*), 47.
Public medical services, A guide to the—A. Faulkner (*Review*), 142.
Pyrexia, 239.

Rectum, Carcinoma of the, 243.
Ridge, Dr. J. J.—Alcohol and public health (*Review*), 138.
Roseola, On epidemic—Dr. C. Dukes (*Review*), 261.

Saint Bartholomew's hospital reports (*Review*), 261.
Saint Thomas's hospital reports (*Review*), 132.
Schuster, Dr.—When may syphilitics marry? (*Review*), 136.
Sciatica—Dr. A. S. Eccles (*Review*), 133.
Science progress (*Review*), 139.
Scraps, 79, 155, 271, 335.
Seborrhœa, On—Dr. J. F. Payne (*Review*), 320.
Senile nerve degeneration, Cases of —S. W. MacIlwaine, 294.
Sex, The human element in—Dr. E. Blackwell (*Review*), 257.
Shoemaker, Dr. J. V. — Materia medica and therapeutics (*Review*), 58.
Sick, Preparations for the, 60, 143, 263, 326.
Simpson, R.—Sciatic neuritis (*Review*), 133.
Skin diseases, Medicated baths in the treatment of—Dr. L. Phillips (*Review*), 258.
Skin, Diseases of the—M. Morris (*Review*), 141.
Skin, Influence of solar rays on the, 245.
Smith, Dr. R. S.—Reports on medicine, 108, 297.
Smith, N.—Spinal caries (*Review*), 313.
Snake-poison, On—Dr. A. Mueller (*Review*), 51.
Snow, Dr. H.—On cancers and the cancer process (*Review*), 128.
Society, Bristol Medico-Chirurgical, 62, 149, 329.
Solar rays on the skin, Influence of, 245.
Somnambulism—Dr. J. M. Clarke, 81.
Spina bifida, 40.
Spinal caries—N. Smith (*Review*), 313.
Spinal lesions, Sensory distribution in, 38.
Spine, Rotary-lateral curvature of the, 37.
Sprains—Dr. C. W. M. Moullin (*Review*), 324.
Squire, Dr. J. E.—The hygienic prevention of consumption (*Review*), 140.
Starr and T. S. Westcott, Drs. L. [Eds.]—An American text-book of the diseases of children (*Review*), 310.
Stewart, W. R. H.—Aids to otology (*Review*), 140.
Sturges, Dr. O.—On chorea (*Review*), 258.
Surgery, Reports on—W. M. Barclay, 37; W. H. Harsant, 242; C. A. Morton, 114; Dr. J. Swain, 302.
Surgery, Text-book of abdominal —S. and G. E. Keith (*Review*), 315.
Sutton, J, B.—Tumours, innocent and malignant (*Review*), 128.
Swain, Dr. J.—Appendicitis, 9; Report on surgery, 302.
Swayne, Dr. J. G.—Ergot of rye as an oxytocic, 225.
Syphilis—E. Cotterell (*Review*), 130.
Syphilis, The treatment of constitutional—Dr. O. Ziemssen (*Review*), 136.
Syphilitics marry?, When may—Dr. Schuster (*Review*), 136.
Syringomyelia, A case of—Dr. F. H. Edgeworth, 100.

Teratologia—Dr. J. W. Ballantyne (*Review*), 137.

Therapeutics, A text-book of general —Dr. W. H. White (*Review*), 49.
Therapeutics, Materia medica and —Dr. J. V. Shoemaker (*Review*), 58.
Thompson, Sir H.—Introduction to the catalogue of the collection of calculi of the bladder (*Review*), 138.
Thorne, Dr. W. B.—The treatment of chronic diseases of the heart by baths and exercises (*Review*), 321.
Thyroid extract in diseases of the skin, 247.
Tooth extraction—J. Gorham (*Review*), 58.
Toxæmia of pregnancy, 119
Toxicology, Forensic medicine and —Dr. J. D. Mann (*Review*), 54.
Traumatic fever, The physiology of death from—Dr. J. D. Malcolm (*Review*), 254.
Trephining in its ancient and modern aspect—Dr. J. F. Horne (*Review*), 311.
Trichorrhexis, 245.
Tubercular pulmonary cavities, Surgical treatment of, 41.
Tumours—J. B. Sutton (*Review*), 128.

Uræmia, Treatment of, 235.
Ureters, Surgery of the, 302.
Urinary organs, Surgical disorders of the—R. Harrison (*Review*), 126.
Urine, A guide to the examination of the—J. W. Legg (*Review*), 133.
Utérus, Traitement chirurgical des affections inflammatoires et néoplasiques de l'—E. Doyen (*Review*), 48.

Vermiform appendix, Pathology of —Dr. T. N. Kelynack (*Review*), 49.
Visiting list, Wright's (*Review*), 325.
Vomiting in pregnancy, 120.

Waldo, Dr. H.—Report on dermatology, 245.
West London Medico-Chirurgical Society, Proceedings of the (*Review*), 135.
Westcott, Drs. L. Starr and T. S. [Eds.]—An American text-book of the diseases of children (*Review*), 310.
White, Dr. W. H.—A text-book of general therapeutics (*Review*), 49.
Will, Dr. J. C. O.—Lectures on genito-urinary diseases (*Review*), 257.
Wilson, Dr. A. M.—Myxœdema (*Review*), 140.
Wilson, Dr. E. T.—Disinfectants and antiseptics (*Review*), 59.
Women, Diseases of—Dr. H. J. Garrigues (*Review*), 250; Dr. A. H. N. Lewers (*Review*), 58; Dr. J. Phillips (*Review*), 52.

Year-book of scientific societies (*Review*), 260.
Year-book of treatment (*Review*), 57.
Yeo, Dr. I. B.—A manual of medical treatment (*Review*), 125.

Ziemssen, Dr. O.—The treatment of constitutional syphilis (*Review*), 136.

### *PUBLICATIONS RECEIVED.*

From THE AMERICAN THERAPIST PUBLISHING COMPANY, New York:
*The American Therapist.* Vol. III., Nos. 1—4. July—Oct., 1894.

From BAILLIÈRE, TINDALL & COX, London:
*Diagrams for Recording Cases of Heart Disease.* By GEORGE HERSCHELL, M.D. 1894.
*Clinical Diagrams with Directions, for Recording Cases of Heart Disease.* By GEORGE HERSCHELL, M.D. 1894.
*Diseases of the Nose.* By JAMES B. BALL, M.D. Second Edition, 1894.
*Hydatid Disease.* Vol. II. By the late JOHN DAVIES THOMAS, M.D. 1894.
*Asthma and Chronic Bronchitis.* By JOHN C. THOROWGOOD, M.D. 1894.
*Urine and Urinary Analysis.* By D. CAMPBELL BLACK, M.D. 1895 (*sic.*)

From JOHN BALE & SONS, London:
*Diseases of the Breast.* By W. ROGER WILLIAMS. 1894.

From BENNETT BROS., LTD., Bristol:
1893. *City and County of Bristol.* Annual Report of the Medical Officer of Health. 1894.

From THE BRITISH MEDICAL TEMPERANCE ASSOCIATION, London:
*The Medical Pioneer.* September, 1894.

From BUREAU D'EXPÉDITION À BUDAPEST DE L'EAU PURGATIVE FRANÇOIS JOSEPH:
*Budapest Souvenir.* 1894.

From CASSELL & CO., LIMITED, London:
*Elements of Surgical Pathology.* By AUGUSTUS J. PEPPER, M.S., M.B. Fourth Edition. 1894.

From THE CHURCH DEFENCE INSTITUTION, London:
*The National Church.* October—December, 1894.

From J. & A. CHURCHILL, London:
*Alpine Climates for Consumption.* By HERBERT JUNIUS HARDWICKE, M.D. 1894.
*Diseases of the Ear and Naso-Pharynx.* By T. MARK HOVELL. 1894.
*The Dynamics of Life.* By W. R. GOWERS, M.D., F.R.S. 1894.

From COMPANIA SUD-AMERICANA DE BILLETES DE BANCO, Buenos Aires:
*Revista de la Sociedad Médica Argentina.* Vol. III., No. 16. 1894.

From CORDES, HERMANNI & CO., Hamburg:
*L'Ichthyol dans le traitement des uréthrites et des cystites.* Par le Doctr. ROBERTO VILLETTI. 1894. [Reprint.]
*Appunti sull 'azione fisiologia e terapeutica dell 'Ittiolo.* Dott. ANGELO CECONI. 1894. [Reprint.]
*Ueber die Anwendung des Ichthyol bei Wunddrunk der Füsse.* Von Dr. LEOPOLD HERZ. 1894. [Reprint.]
*Psorospermoses Cutis.* Von Dr. A. RAVOGLI. 1894. [Reprint.]
*Ueber die Beziehungen des Ekzems zu den Schleimhäuten.* Von. Dr. von SEHLEN. 1894. [Reprint.]

From THE F. A. DAVIS COMPANY, Philadelphia. (Per F. J. REBMAN, London):
*Syllabus of Lectures on Embryology.* By WALTER PORTER MANTON, M.D. 1894.
*Practical Uranalysis and Urinary Diagnosis.* By CHARLES W. PURDY, M.D. 1894.
*Text-book of Hygiene.* By GEORGE H. ROHÉ, M.D. Third Edition. 1894.

From ESTABLECIMIENTO TIPOGRÁFICO "SUCESORES DE RIVADENEYRA," Madrid:
*El Eco del Consultorio.* Año I., Núm. 2. 15 de Noviembre de 1894.

From FANNIN & CO., Dublin:
*Diseases and Deformities of the Spine.* By R. L. SWAN. 1894.

From GOVERNMENT PRINTING OFFICE, Washington:
*Index-Catalogue of the Library of the Surgeon-General's Office, United States Army.* Vol. XV. Universidad-Vzoroff. 1894.

From "THE ILLUSTRATED LONDON NEWS" OFFICE, London:
*The English Illustrated Magazine.* November, 1894.

From R. JACKSON, Leeds:
*Ethics in Medicine in Relation to Unorthodox Physic.* By a West Riding Practitioner. 1894.

From THE JOHNS HOPKINS PRESS, Baltimore:
*The Johns Hopkins Hospital Reports.* Vol. IV., No. 6. *Report in Surgery,* II. 1894.

From DR. H. MACNAUGHTON JONES, London:
*Clinical Papers.* By H. MACNAUGHTON JONES, M.D. [Reprints.]

From 723 KANSAS AVENUE, Topeka, Kansas:
*The Kansas Medical Journal.* Vol. VI., Nos. 34, 37, 43. August 25th, September 15th, October 27th. 1894.

From H. K. LEWIS, London:
*Medical Nursing.* By the late JAMES ANDERSON, M.D. Edited by ETHEL F. LAMPORT. 1894.
*The Dyspepsia of Phthisis.* By W. SOLTAU FENWICK, M.D. 1894.
*On Gangrene of the Limbs following Typhoid Fever.* By F. DAWTREY DREWITT, M.D. 1894.
*The Theory and Practice of Medicine.* By FREDERICK T. ROBERTS, M.D. Ninth Edition. 1894.
*Enlargement of the Prostate.* By C. W. MANSELL MOULLIN, M.A., M.D. 1894.

From METHUEN & CO., London:
*The Vaccination Question.* By ARTHUR WOLLASTON HULTON, M.A. 1894.

From OLIVER & BOYD, Edinburgh:
*The Disorders of Speech.* By JOHN WYLLIE, M.D. [1894.]

From YOUNG J. PENTLAND, Edinburgh:
*Prescribing and Treatment for Infants and Children.* By PHILIP E. MUSKETT. Third Edition. 1894.
*First Aid to the Injured and Management of the Sick.* By E. J. LAWLESS, M.D. 1894.

From G. P. PUTNAM'S SONS, New York:
*A Clinical Manual.* By ANDREW MACFARLANE, M.D. 1894.

From JOHN MORGAN RICHARDS, London:
*Medical Reprints.* June—November, 1894.
*Aluminium Pocket Calendar.* 1895.

From "THE PROVINCIAL MEDICAL JOURNAL," Leicester:
*Abdominal Section.* By W. J. STEWART MCKAY, M.B. 1894. [Reprint.]

From DR. HUNTER ROBB, Baltimore:
*The Limitations of the Use of the Pessary.* By HUNTER ROBB, M.D. 1894. [Reprint.]
*Abstract of Two Articles treating of Progress in Midwifery.* By HUNTER ROBB, M.D. 1894. [Reprint.]
*Notes on Gynæcological Technique.* By HUNTER ROBB, M.D. 1894. [Reprint.]
*Asepsis in Minor Procedures.* By HUNTER ROBB, M.D. 1894. [Reprint.]

From 4 RUE ANTOINE-DUBOIS, Paris:

*La Médecine hypodermique.* 7e Année. No. 15. 5 Août, 1894.

From RUSSISCHE PHARMACEUTISCHE HANDELGESELLSCHAFT, St. Petersburg:
*Sperminum-Poehl in chemischer, physiologischer und therapeutischer Beziehung.* Von Dr. G. BUBIS. 1894.

From W. B. SAUNDERS, Philadelphia:
*Essentials of Diseases of Eye, Nose, and Throat.* By EDWARD JACKSON, M.D. and E. B. GLEASON, M.D. Second Edition. 1894.
*Essentials of Diseases of the Ear.* By E. B. GLEASON, M.D. 1894.
*Manual of Human Physiology.* By JOSEPH H. RAYMOND, M.D. 1894.

From W. B. SAUNDERS, Philadelphia *(continued)*:
*Manual of Modern Surgery.* By JOHN CHALMERS DA COSTA, M.D. 1894.
*Temperature Chart.* Prepared by D. T. LAINÉ, M.D. 1894.

From THE SCIENTIFIC PRESS, LIMITED, London:
*Medical History from the Earliest Times.* By EDWARD THEODORE WELLINGTON, M.B. 1894.

From AUG. SIEGLE, London:
*The Therapist.* Vol. IV., No. 5. 15th May, 1894.

From SMITH, ELDER & CO., London:
*Essays on State Medicine.* I. Compulsory Vaccination and Small-Pox in Relation to Vaccination. By ERNEST HART, D.C.L. 1894. II. Compulsory Notification in England and Wales in 1892. III. State Aid in Relation to Port Cholera Expenses. By ERNEST HART, D.C.L. 1894.
*On the Use of Opium in India:* A Summary . . . prepared by ERNEST HART, D.C.L. 1894.
*The Nurseries of Cholera: its Diffusion and its Extinction.* By ERNEST HART, D.C.L. 1894.

From G. STEINHEIL, Paris:

*De l'Agrandissement Momentané du Bassin.* Par ADOLPHE PINARD. 1894.

From STEPHENS & EYRE, Bristol:
*What I Saw at the Pasteur Institute.* By T. A. WILLIAMS. N.D. [Reprint.]

From Dr. D. D. STEWART, Philadelphia:
*Further Remarks on the occurrence of a Form of Non-Albuminous Nephritis other than Typical Fibroid Kidney.* By D. D. STEWART, M.D. 1894. [Reprint.]
*A Serious Fallacy attending the Employment of certain Delicate Tests for the Detection of Serum-Albumin in the Urine, especially the Trichlor-acetic Acid Test.* By D. D. STEWART, M.D. 1894. [Reprint.]

From STICKLAND & CO., London:
*Improved Sick-Room Chart.*

From TIPOGRAFÍA HISPANO-AMERICANA, Barcelona:
*Caracteristica terapéutica y Especialización clínica de las Aguas termales de La Garriga.* Por el Dr. M. MANZANEQUE. 1894.

From UNION MISSION AND HOSPITAL, Philadelphia:
*Union Mission Hospital Reports, Philadelphia.* May, 1894.

From WHITTAKER & CO., London:
*Dissections Illustrated.* By C. GORDON BRODIE. Part III. [1894.]

From WILLIAMS & NORGATE, London:
*Teratologia.* By J. W. BALLANTYNE, M.D. Nos. 1, 2. April, July, 1894.

From Dr. H. AUGUSTUS WILSON, Philadelphia:
*Bone Operations for the Correction of Club-foot, based upon an Analysis of* 435 *Operations by* 108 *Operators.* By H. AUGUSTUS WILSON, M.D. 1893. [Reprint.]
*Teno-Suture and Tendon Elongation and Shortening by Open Incision.* By H. AUGUSTUS WILSON, M.D. [N.D.] [Reprint.]
*Can Physicians Honorably Accept Commissions from Orthopedic Instrument Makers?* By H. AUGUSTUS WILSON, M.D. 1894. [Reprint.]

From JOHN WRIGHT & CO., Bristol:
*Myxœdema, Cretinism and the Goitres.* By EDWARD T. BLAKE, M.D. 1894.
*Wright's Improved Visiting List.* Compiled by ROBERT SIMPSON. 1895.
*Diseases of the Upper Respiratory Tract: The Nose, Pharynx and Larynx.* By P. WATSON WILLIAMS, M.D. [1894.]

From J. YOUNG & SONS, Perth:
*The Sixty-Seventh Annual Report of James Murray's Royal Asylum, Perth.* 1894.

